# Antimicrobial resistance, pathogenic potential, and genomic features of carbapenem-resistant *Klebsiella pneumoniae* isolated in Chile: high-risk ST25 clones and novel mobile elements

**DOI:** 10.1128/spectrum.00399-23

**Published:** 2023-09-14

**Authors:** Marcelo Veloso, Patricio Arros, Joaquin Acosta, Roberto Rojas, Camilo Berríos-Pastén, Macarena Varas, Pamela Araya, Juan Carlos Hormazábal, Miguel L. Allende, Francisco P. Chávez, Rosalba Lagos, Andrés E. Marcoleta

**Affiliations:** 1 Grupo de Microbiología Integrativa, Laboratorio de Biología Estructural y Molecular BEM, Departamento de Biología, Facultad de Ciencias, Universidad de Chile, Santiago, Chile; 2 Instituto de Salud Pública, Santiago, Chile; 3 Millennium Institute Center for Genome Regulation (CGR), Facultad de Ciencias, Universidad de Chile, Santiago, Chile; 4 Laboratorio de Microbiología de Sistemas, Departamento de Biología, Facultad de Ciencias, Universidad de Chile, Santiago, Chile; Universidade de São Paulo, São Paulo, Brazil

**Keywords:** carbapenem-resistant *Klebsiella pneumoniae*, antibiotic resistance, virulence, genomic surveillance, plasmids, genomic islands, South America

## Abstract

Multidrug- and carbapenem-resistant *Klebsiella pneumoniae* (CR-*Kp*) are critical threats to global health and key traffickers of resistance genes to other pathogens. Despite the sustained increase in CR-*Kp* infections in Chile, few strains have been described at the genomic level, lacking details of their resistance and virulence determinants and the mobile elements mediating their dissemination. In this work, we studied the antimicrobial susceptibility and performed a comparative genomic analysis of 10 CR-*Kp* isolates from the Chilean surveillance of carbapenem-resistant *Enterobacteriaceae*. High resistance was observed among the isolates (five ST25, three ST11, one ST45, and one ST505), which harbored 44 plasmids, most carrying genes for conjugation and resistance to several antibiotics and biocides. Ten plasmids encoding carbapenemases were characterized, including novel plasmids or variants with additional resistance genes, a novel genetic environment for *bla*_KPC-2_, and plasmids widely disseminated in South America. ST25 K2 isolates belonging to CG10224, a clone traced back to 2012 in Chile, which recently acquired *bla*_NDM-1_, *bla*_NDM-7_, or *bla*_KPC-2_ plasmids stood out as high-risk clones. Moreover, this corresponds to the first report of ST25 and ST45 *Kp* producing NDM-7 in South America and ST505 CR-*Kp* producing both NDM-7 and KPC-2 worldwide. Also, we characterized a variety of genomic islands carrying virulence and fitness factors. These results provide baseline knowledge for a detailed understanding of molecular and genetic determinants behind antibiotic resistance and virulence of CR*-Kp* in Chile and South America.

**IMPORTANCE:**

In the ongoing antimicrobial resistance crisis, carbapenem-resistant strains of *Klebsiella pneumoniae* are critical threats to public health. Besides globally disseminated clones, the burden of local problem clones remains substantial. Although genomic analysis is a powerful tool for improving pathogen and antimicrobial resistance surveillance, it is still restricted in low- to middle-income countries, including Chile, causing them to be underrepresented in genomic databases and epidemiology surveys. This study provided the first 10 complete genomes of the Chilean surveillance for carbapenem-resistant *K. pneumoniae* in healthcare settings, unveiling their resistance and virulence determinants and the mobile genetic elements mediating their dissemination, placed in the South American and global *K. pneumoniae* epidemiological context. We found ST25 with K2 capsule as an emerging high-risk clone, along with other lineages producing two carbapenemases and several other resistance and virulence genes encoded in novel plasmids and genomic islands.

## INTRODUCTION

Multidrug-resistant (MDR) *Klebsiella pneumoniae*, particularly carbapenem-resistant *Klebsiella pneumoniae* (CR-*Kp*) strains causing high mortality and morbidity, are critical concerns (1). These high-risk pathogens have developed mainly by acquiring different mobile genetic elements (MGEs), including plasmids and genomic islands (GIs) encoding an array of virulence and antibiotic resistance factors (2).

Carbapenems are last-resort antibiotics for treating MDR *Enterobacteriaceae* infections (3). The most concerning carbapenem resistance factors are carbapenem-inactivating beta-lactamases, especially the *K. pneumoniae* carbapenemase (KPC) distributed worldwide. Moreover, this pathogen is a key trafficker of other carbapenemases, including NDM, VIM, and OXA-48, all encoded in MGEs (1, 2, 4).

In Chile, after identifying the first introduced KPC-producing *K. pneumoniae* infection in 2012, the Chilean Public Health Institute (ISP) established clinical surveillance of carbapenem resistance in *Enterobacteriaceae* isolated in clinical settings. Between 2014 and 2019, ISP received 7,227 carbapenem-resistant isolates from different human sources, of which 2,225 (30.7%) were carbapenemase-positive, increasing from 15 isolates in 2014 to 1,303 in 2019. Eighty-three percent of these positive isolates were collected from hospitals in the Metropolitan region, followed by 3% from the Los Lagos region. Fifty-eight percent of the isolates corresponded to *Klebsiella* spp., while the predominant carbapenemases corresponded to KPC (74%), NDM (11%), and VIM (14%) (https://vigilancia.ispch.gob.cl/app/iaas). Furthermore, between 2012 and 2020, a sustained increase in *K. pneumoniae* resistance to meropenem, ertapenem, and imipenem was observed (5).

Despite the increasing incidence of CR-*Kp* infections in Chile, little is known about these strains’ genomic and molecular features, their phylogenetic relationships with the South American and global *K. pneumoniae* population, and the mobile elements mediating dissemination of virulence and resistance genes. Furthermore, public databases lack complete genome sequences of CR-*Kp* isolated in Chile, hampering their inclusion in population genomics studies to place them in a global epidemiological context.

This work characterized 10 CR-*Kp* strains from the Chilean CR *Enterobacteriaceae* surveillance, determining their phenotypic antibiotic resistance, complete genome sequence, virulence and resistance gene profile, and MGEs associated with these factors. Our results provide valuable baseline knowledge of *K. pneumoniae* causing infections in Chile and the mobile genetic platforms that could mediate virulence and resistance gene dissemination in our regional context.

## MATERIALS AND METHODS

### Isolate collection and antimicrobial susceptibility tests

The 10 CR-*Kp* isolates described in this study were collected by ISP in 2018 and 2019 as part of the national surveillance of carbapenem-resistant *Enterobacteriaceae* (Table S1). Antimicrobial susceptibility testing was performed by broth microdilution and epsilometry (E-test), following the M100 Performance Standards for Antimicrobial Susceptibility Testing, 31st edition. Tigecycline resistance was interpreted according to EUCAST 2019 guidelines. The Blue Carba and boronic acid tests and the molecular detection of carbapenemases were made following standard procedures conducted at ISP (6).

### Genome sequencing

Genomic DNA was extracted using the GeneJET Kit (Thermo Scientific) and quantified using a Qubit fluorimeter (Invitrogen). Illumina sequencing (100 bp paired-end) was performed by Macrogen Inc. (Korea) using the TruSeq Nano DNA kit and a Hiseq4000 machine. FastQC v0.11.9 (7) and Trimmomatic v0.36 (8) were used for quality check and trimming. Nanopore libraries prepared with the Native Barcoding Expansion (EXP-NBD104) and the 1D Ligation Sequencing (SQK-LSK109) kits were run on a MinION device and FLO-MIN106 (R9.4) flow cells. After base-calling with Guppy v5.0.7, the raw nanopore reads were corrected, trimmed, and assembled using Canu v2.3 (9). Hybrid assemblies were performed using Unicycler v0.4.9b (10) with the short and long reads plus the Canu assembly as input. The assemblies were evaluated using QUAST v5.0.2 (11) and CheckM (12), and annotated with RAST v2.0 (13).

### *K. pneumoniae* genome database construction and phylogenomic analyses

All the available (17,612) *K. pneumoniae* species complex (*Kp*SC) genomes were downloaded from the NCBI RefSeq database on April 5, 2023. Genomes of poor quality, having ambiguous nucleotide bases, and non-standard *Klebsiella* genomes were filtered out following the criteria proposed by Hennart et al. (2022) (14) (≥1,000 contigs, genomic size ≤4.5 Mbp or ≥6.5 Mbp, >59% GC content, <96% ANI to reference KpSC genomes). The resulting 11,877 genomes were screened for MLST, species, and other relevant information using Kleborate v2.3.2 (15). Only Kp1 genomes were selected, corresponding to 10,904 isolates, including the 10 genomes described in this study.

The 629 loci cgMLST scheme recently proposed for *K. pneumoniae*, scgMLSTv2 (14) was used to search for alleles in all the Kp1 genomes using BLASTn v2.13.0, with 95% identity and 95% coverage thresholds. The best hit for each allele was chosen according to the bitscore. Then, the selected alleles were used to generate cgMLST profiles for each genome. Ninety-four Kp1 genomes were discarded due to missing allele data (more than 30 loci were missing), generating a final set of 10,810 Kp1 genomes. From these, a total of 602 were identified as being from South America.

The cgMLST profiles of the 10,810 Kp1 genomes and 34,055 reference profiles were used as input for the LINcoding algorithm (16) to assign cgLIN codes to each genome, as specified by Hennart et al. (14). The assigned cgLIN codes were used to identify clonal groups and sublineages. Based on the cgLIN codes, 403 closest neighbors of the assembled Chilean genomes were selected to build a minimum spanning tree (MST). cgMLST profiles and metadata (country of origin and collection year) of selected genomes were used as input for the PHYLOViZ Online platform (17) to compute allelic distances between all selected genomes and construct the MST.

A core genome multiple sequence alignment (cg-MSA), including the 629 loci from the scgMLSTv2 scheme, was constructed using MAFFT v7.310 for the 602 South American *K. pneumoniae* genomes. Then, this alignment was used to infer a phylogenetic tree, using IQ-TREE v2.2.2.3, with a seed of 890.997, the GTR+G model, and 1,000 bootstrap iterations. Finally, the tree obtained was formatted and complemented with relevant metadata using iTOL (18).

### Identification of resistance and virulence genes

ARG prediction was made with RGI v5.2.0 (CARD database v3.1.4) (19) and AMRFinderPlus v3.10.18 (20), harmonizing and consolidating both outputs using hAMRonization v1.0.3 (https://github.com/pha4ge/hAMRonization), following ARG classification according to the CARD ontology. Kleborate ARG prediction was also considered. Metal and biocide resistance gene identification was performed using Diamond blastX (21) to compare the genomes of the isolates with the BACMET database of experimentally confirmed resistance genes v2.0 (22). Ninety percent of identity and coverage were used to consider true positives. Virulence factors were predicted using Kleborate, VFanalyzer, and the VFDB Database (23). The heat maps showing resistance and virulence genes were generated using iTOL.

### MGE analyses

The identification, clustering, and typing of the plasmids from the isolates described in this work were made using PlasmidVerify (24) and the MOB-suite v3.1.4 mob_typer module (25) with default parameters. Additionally, the mob_recon module was used to reconstruct and type the plasmids in the 602 South American *K. pneumoniae* genomes. Transfer origins (oriT) and conjugation genes were identified using oriTfinder (26). Integrons and insertion sequences were predicted using IntegronFinder v1.5.1 (27) and ISfinder (28). tDNA classification and prediction of integrated MGEs were performed using our tool Kintun-VLI (https://github.com/GMI-Lab/Kintun-VLI). Prophage identification was made using PHASTER (29). MGE clustering to define non-redundant GIs and prophages was performed with CD-HIT (30) (≥80% identity and ≥85% coverage).

## RESULTS

### Phenotypic AMR of CR-*Kp* causing infections in Chile

We studied the genomic features, antimicrobial resistance, virulence potential, and MGEs of CR-*Kp* causing infections in Chile. For this, we selected 10 isolates from the ISP’s CR *Enterobacteriaceae* surveillance for complete genome sequencing and phenotypic characterization based on the following criteria: (i) confirmed high levels of carbapenem resistance and species identification as *K. pneumoniae*, (ii) to proportionally cover the three main regions of the country that sent CR-*Kp* isolates to ISP (Metropolitan, Los Lagos, and Maule), (iii) for showing multidrug resistance and a different antibiotic susceptibility profile, and (iv) to cover different infected tissues from patients of varying ages and genders (Table S1).

MIC determinations indicated that all of them were resistant to third- and fourth-generation cephalosporins (most also to combinations with beta-lactamase inhibitors) and to meropenem and ertapenem, while seven were resistant to imipenem (Table 1). PCR analysis showed either NDM or KPC carbapenemase genes in six strains, while two encoded both and two lacked these genes (Table S2). Furthermore, phenotypic tests confirmed carbapenemase production in the positive isolates, while ESBL production was detected in all of them. Additionally, most were resistant to ciprofloxacin, sulfamethoxazole, and gentamicin, while one showed resistance to amikacin, two to tigecycline, and two to colistin. Thus, the Chilean isolates corresponded to extensively drug-resistant *K. pneumoniae* with high resistance to carbapenems and other last-resort antimicrobial drugs.

**TABLE 1:** Minimum inhibitory concentrations for different antibiotics^*a*^

Isolate	FEP	CAZ	CRO	CZA	C/T	TZP	ETP	IPM	MEM	TGC	CIP	AMK	GEN	SXT	CST
VA4	>16*	>16*	>4*	ND	ND	>256/4*	>1*	>8*	>32*	1*	>2*	≤4	>8*	≥4/76*	≤1
VA32	>16*	>16*	>4*	2/4	>32/4*	>256/4*	>1*	>8*	>32*	0.25	>2*	≤4	≤0.5	≥4/76*	≤1
VA126	>256*	>256*	>32*	ND	ND	>256/4*	16*	0.25	4*	0.5	>2*	≤4	≤0.5	≥4/76*	≤2
VA172	>16*	>16*	>32*	1/4	>32/4*	>256/4*	4*	2*	2*	0.5	>2*	≤4	≤0.5	≥4/76*	4*
VA564	>16*	>16*	>32*	4/4	>32/4*	>256/4*	4*	0.5	4*	≤0.25	>2*	≤4	>8*	≥4/76*	≤0.25
VA569	>16*	>16*	>32*	16/4*	>32/4*	>256/4*	>32*	>8*	>8*	≤0.25	>2*	≤4	>8*	≥4/76*	≤0.25
VA591	>16*	>16*	>32*	ND	ND	128/4*	32*	>8*	8*	0.5	2*	≤4	>8*	≤1/19	>4*
VA681	>16*	>16*	>32*	≤0.5/4	>32/4*	128/4*	8*	≤1	4*	0.5	>2*	≤4	>8*	≥4/76*	≤0.25
VA684	>16*	>16*	>32*	>16/4*	>32/4*	64/4*	4*	8*	8*	0.5	≤0.064	≤4	≤0.5	≥4/76*	≤0.25
VA833	>16*	>16*	>32*	>16/4*	>32/4*	>256/4*	>3*	>8*	>8*	2*	>2*	>32*	>8*	≥4/76*	≤0.25

^*a*^ FEP, cefepime; CAZ, ceftazidime; CRO, ceftriaxone; CZA, ceftazidime/avibactam; C/T, ceftolozane/tazobactam; TZP, piperacillin/tazobactam; ETP, ertapenem; IPM, imipenem; MEM, meropenem; TGC, tigecycline; CIP, ciprofloxacin; AMK, amikacin; GEN, gentamicin; SXT, sulfamethoxazole; CST, colistin; *, resistant.

### Genomic features of Chilean CR-*Kp* and relationships with *K. pneumoniae* isolated worldwide

**Fig 1:**
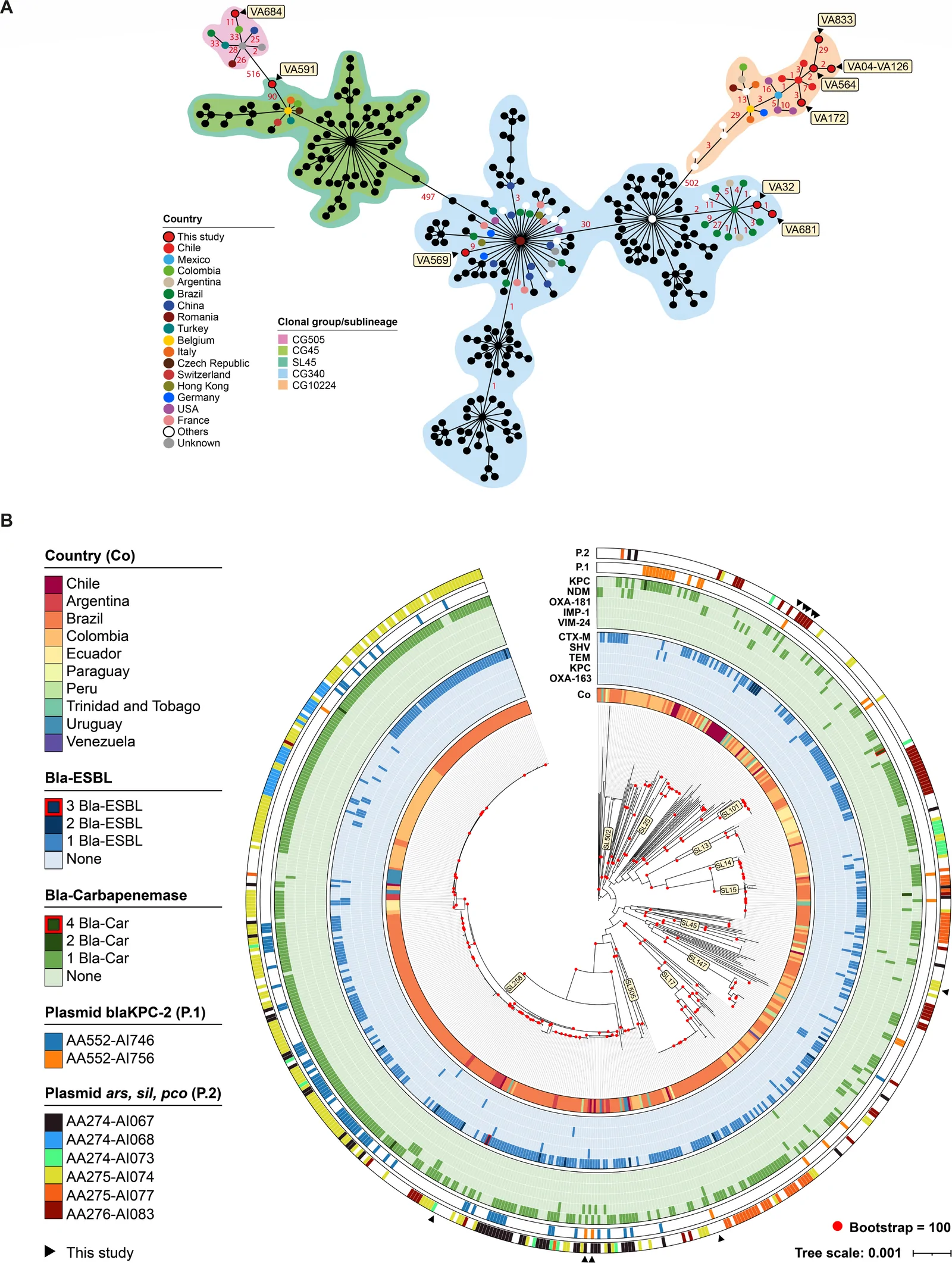
Phylogenomic relationships between Chilean CR-*Kp* isolates and *K. pneumoniae* isolated worldwide. (**A**) Minimum spanning tree including the Chilean isolates described in this study and 403 *Kp*1 genomes from our global data set, belonging to the closest lineages according to the cgMLST analysis. Selected isolates located in the surroundings of the Chilean isolates were colored according to their geographical origin. The numbers on the edges indicate the allelic distances. (**B**) Phylogenetic tree of 602 *K*. *pneumoniae* strains isolated in South America, including the 10 isolates described in this study. The tracks correspond to (inner to outward) the country of origin, ESBL content, carbapenemase content, and the presence of defined KPC-2 or metal resistance plasmids.

We determined the complete genome sequence of each isolate by combining Illumina and Nanopore data, obtaining a closed chromosome and two to six plasmids (Table 2). According to Kleborate analysis, all belonged to the *K. pneumoniae sensu stricto* (*Kp*1) species, five ST25, three ST11, one ST45, and one ST505, referred to the classical seven-gene MLST scheme. To investigate the relationships of these strains with *K. pneumoniae* isolated worldwide, we constructed a database including a total of 10,810 *Kp*1 genomes (Table S3) and performed a 629-gene cgMLST analysis following the method recently proposed by Hennart et al. (14). Based on this classification, we identified the sublineages (SL, threshold: 190 allelic mismatches) and clonal groups (CG, threshold: 43) encompassing the Chilean isolates, selecting the close neighbors to construct a MST (Fig. 1A).

**TABLE 2:** Genome assembly stats

Strain	ST	Total length (bp)	%GC	Cov. depth	Compl. (%)	Contam. (%)	Chromosome length (bp)	N° plasmids	Plasmids length (kbp)	Accession
VA04	ST25	5,762,453	56.98	176X	99.90	0.90	5,456,744	4	6^*a*^; 46; 58; 196	CP093501–CP093505
VA32	ST11	5,731,565	57.17	202X	100.00	0.08	5,286,512	5	7; 58; 74; 105; 200	CP093495–CP093500
VA126	ST25	5,757,426	56.98	181X	92.28	0.90	5,121,870	4	12; 42; 68; 175	CP093489–CP093494
VA172	ST25	5,403,938	57.44	198X	100.00	0.39	5,246,520	2	67; 90	CP093486–CP093488
VA564	ST25	5,718,217	57.00	182X	100.00	1.21	5,406,546	4	33; 37; 63; 179	CP093481–CP093485
VA569	ST11	5,713,876	57.13	164X	100.00	0.59	5,584,676	4	6; 7; 35^*a*^; 78	CP093476–CP093480
VA591	ST45	5,653,497	57.18	180X	100.00	0.08	5,430,590	3	9; 46; 160	CP093471–CP093475
VA681	ST11	5,554,568	57.14	204X	99.63	0.39	5,250,844	5	5; 14; 36; 58; 191	CP093465–CP093470
VA684	ST505	5,818,826	56.74	195X	100.00	0.08	5,176,823	7	2; 6; 8; 37^*a*^; 49; 146; 388	CP093458–CP093464
VA833	ST25	5,997,469	56.80	220X	99.74	1.25	5,458,013	6	7; 45^*a*^; 56^*a*^; 92; 165; 176	CP093451–CP093457

^*a*^ Incomplete plasmids.

The three ST11 isolates were grouped with strains from the globally disseminated SL258, which comprises most CR-*Kp* isolated worldwide and is highly represented in our database (3,554 of 10,810 genomes). More specifically, they belonged to CG340, the third more frequent of this SL in our data set, following CG258 and CG11. VA569 grouped with strains from diverse origins, while VA32 and VA681 formed a more distant group, including several isolates from Brazil and Argentina. The other isolates covered less frequent and disseminated lineages, such as SL45 (213 genomes), where VA591 formed a CG apart from CG45 and the other genomes, corresponding to a novel CG not reported previously. Also, SL505 (eight genomes), where VA684 clustered close to a Colombian strain isolated in 2011, and with more distant isolates from China (2011), Romania (2013), Turkey (2013), and Brazil (2020).

Meanwhile, the five ST25 isolates belonged to SL25 (81 genomes), specifically to CG10224, one of the two CGs from this SL with CG25. Except for the slightly more divergent VA833, the other four ST25 isolates clustered very close to Chilean isolates previously deposited in the database, including two collected in 2012 and one in 2013. These strains are the older CG10224 in our data set and were very similar to an isolate from Mexico in 2013 and another Chilean isolate from 2017. Thus, the CG10224 ST25 clone would have a relatively narrow distribution with a sustained presence in Chile, at least since 2012.

To place the Chilean isolates in a continental CR-*Kp* landscape, we identified 602 genomes from our data set coming from South America (Table S4) and selected them to construct a cg-MSA including the 629 loci from the scgMLSTv2 scheme and infer a distance tree (Fig. 1B). These isolates came from Brazil (337), Colombia (166), Chile (21), Argentina (20), and other less-represented countries. Therefore, we observed over 70 SLs, indicating a remarkable diversity of *K. pneumoniae* lineages circulating in South America. A strong presence of SL258 was observed in most countries, with CG340 predominating in Brazil, Argentina, and Chile and CG258 in Colombia, Ecuador, and Uruguay. On the other hand, SL25-CG10224 was found in Argentina, Chile, and Colombia, while SL25-CG25 was found in Brazil and Peru. Only one SL505-CG505 genome was found in Chile, Brazil, and Colombia.

Regarding beta-lactamase gene content, 70% of the South American isolates encoded at least one carbapenemase, predominating *bla*_KPC-2_ (73% of the carbapenemase-positive isolates), followed by *bla*_KPC-3_ (15%) and *bla*_NDM-1_ (8%) (Fig. 1B; Fig. S1). Additionally, 13 isolates had two carbapenemase genes (mainly KPC-2 plus NDM-1), including VA684 (ST45) and VA833 (ST25). Moreover, 62% of the isolates carried one or more acquired ESBLs, mostly *bla*_CTX-M-15_ (53%), *bla*_CTM-X-14_ (15%), *bla*_CTX-M-2_ (15%), and *bla*_SHV-12_ (5%) with the sporadic presence of *bla*_SHV-12_, *bla*_TEM-26_, and *bla*_TEM-15_. Remarkably, of the nine Chilean isolates belonging to SL25-CG10224, only those described in this study carried carbapenemase genes, while those isolated between 2012 and 2017 lacked these genes. Moreover, the isolates collected in 2017, 2018, and 2019 tended to host an increased load of ESBLs, suggesting a more recent acquisition of carbapenemases and extra ESBLs in this CG in Chile. Additionally, VA591 was the only SL45 South American isolate encoding carbapenemases.

### Genome-wide resistance gene profile

**Fig 2:**
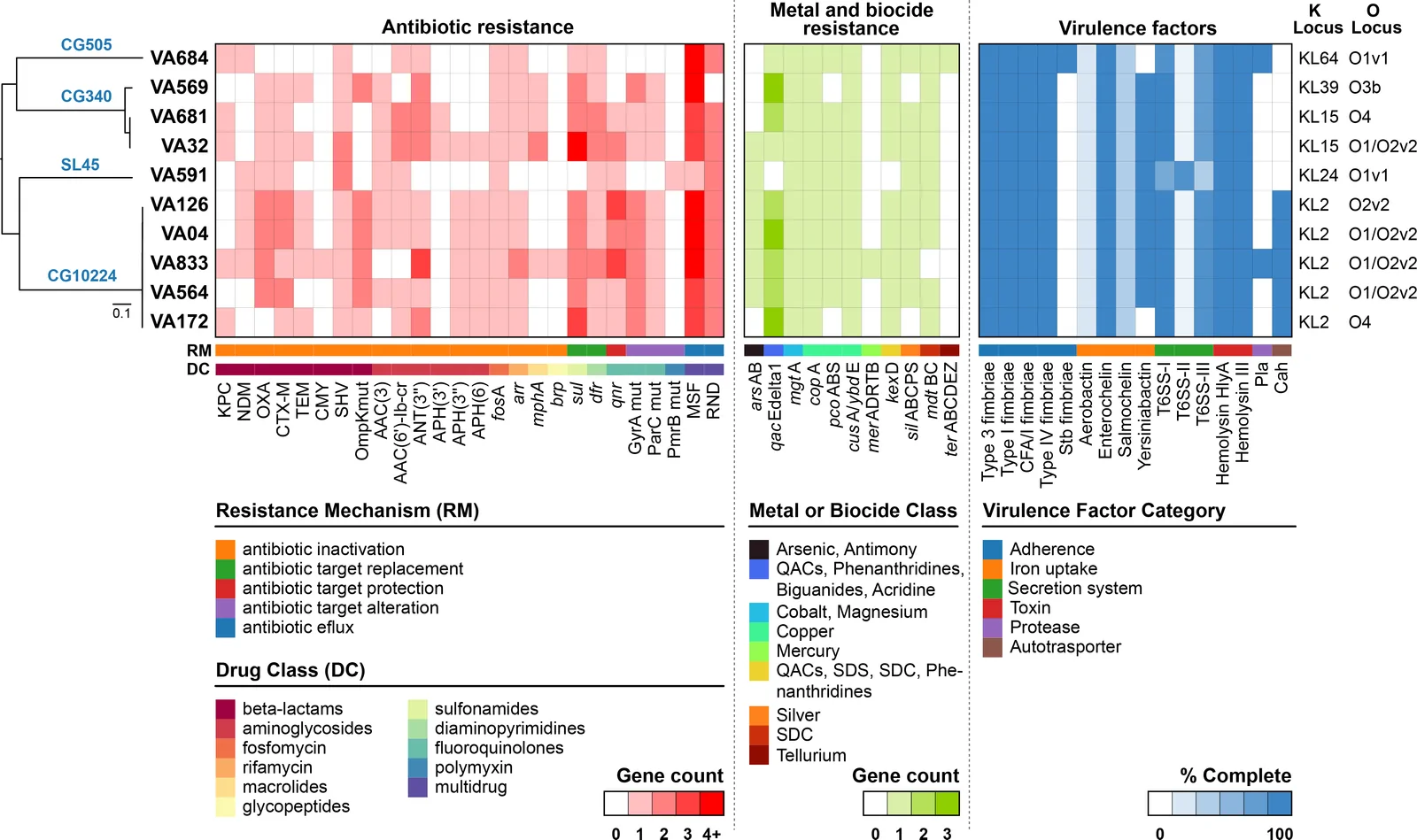
Genome-wide resistance and virulence gene profile of the CR-*Kp* isolates described in this study. Bioinformatic prediction and categorization of genes conferring resistance to antibiotics (left panel), resistance to metals and other biocides (middle panel), and virulence factors (right panel). For resistance genes, the heat map color scale indicates the absolute number of genes found per genome in each category. For virulence genes, the heat map colors represent the percentage of genes involved in the respective virulence trait found in each isolate over the number of known genes required to express that trait. The tree shown was inferred based on a cg-MSA analysis including the 629 loci of the scgMLSTv2 scheme.

We performed a genome-wide search for resistance genes to investigate the complete resistome present in the Chilean isolates. Following the high experimental beta-lactam resistance, we identified up to eight *bla* genes per isolate (Fig. 2; Table S5), three carrying *bla*_KPC-2_, and one *bla*_KPC-2_+*bla*_NDM-1_, a relatively rare combination. Moreover, three isolates had *bla*_NDM-7_, a significantly less frequent variant of NDM-1 with increased activity, while VA684 (CG505) carried *bla*_NDM-7_+*bla*_KPC-2_, a rare combination present only in other two distant genomes (CG340) from Brazil (Fig. 1B).

Additionally, we identified *bla*_CTX-M-15_ and the less frequent *bla*_CTX-M-2_ and *bla*_CTX-M3_ ESBLs (Fig. S1), including the rare combinations *bla*_CTX-M-15_+*bla*_CTX-M-2_ and *bla*_CTX-M-15_+*bla*_SHV-31_. Other acquired beta-lactamases included CMY-2, an ampC beta-lactamase rarely found in *K. pneumoniae* that was absent in the other South American isolates. Additionally, six isolates had mutations in the OmpK35 and OmpK36 porins, which would account for the carbapenem resistance in VA564 and VA569 lacking carbapenemase genes.

Besides, several aminoglycoside resistance genes were found, including several variants of aminoglycoside nucleotidyltransferases (ANTs), phosphotransferases (APHs), and acetyltransferases (AACs), including AAC(6′)-lb-cr reported to confer fluoroquinolone resistance (31) (Fig. 2, left panel) (Table S5). Accordingly, over half of the isolates resisted gentamicin (Table 1). On the other hand, VA833 carried the *armA* gene encoding a 16S rRNA guanine(1405)-*N*(7)-methyltransferase, which could account for its unusually high amikacin resistance (Table 1), as reported previously in *K. pneumoniae* (32).

Regarding fluoroquinolone resistance, most isolates encoded the *adeF* efflux pump, up to four quinolone resistance proteins (mainly QnrB1 and QnrB19), and carried one or two point mutations in GyrA (mainly 83Y, 83I, 87G, and 87N) and the ParC-80I mutation. These findings correlated with ciprofloxacin resistance in most isolates, while the absence of acquired resistance genes and mutations could explain VA684 sensitivity.

Although VA172 and VA591 showed colistin resistance, none carried *mcr* genes. Therefore, we searched for known mutations conferring resistance to this antibiotic in *K. pneumoniae* (33). In this regard, VA591 had the PmrB T157P mutation, while VA172 showed no mutations in all the genes examined, including PmrA, PmrB, MgrB, PhoP, PhoQ, and CrrB. However, in this last isolate, we identified an IS1 insertion sequence integrated into the −39 position of the *mgrB* promoter region, which is a possible explanation for the colistin resistance in this strain. Conversely, we could not find *tet(X*) genes or mutations in ArnR, AcrR, and RpsJ previously linked to tygecycline resistance in *K. pneumoniae* (34). Thus, no clear genetic bases could be established for the high resistance to this antibiotic shown by VA04 and VA833.

Among other resistance determinants found in the Chilean strains, mainly in VA833, are the macrolide 2′-phosphotransferase MPH(2′)-I and a BRP(MBL) bleomycin resistance protein. On the other hand, most isolates encoded several genes involved in resistance to metals and biocides, including copper, arsenic, silver, mercury, tellurium, acridine, halogens, phenolic and quaternary ammonium compounds, and bile salts (Fig. 2, middle panel).

### Virulence factors present in Chilean CR-*Kp*

Next, we identified and categorized the virulence factors present in the Chilean CR-*Kp* (Fig. 2, right panel). The five ST25 isolates displayed a K2 capsule associated with increased virulence (2), while the ST11 isolates had KL15 and KL39. The other isolates had KL24 (ST45) and KL64 (ST505). In terms of adhesion factors, all had the *fim*, *mrk*, *ecp*, and *pil* operons, encoding a type 1 (T1P) fimbriae, a type 3 (MR/K) pili, a homolog of the *Escherichia coli* common pilus (ECP), and a type 4 hemorrhagic *E. coli* pilus (HCP), respectively, all of them which were reported to be involved in cell adherence to tissues (35). Further, VA684 has a homolog of the type 4 *stb* fimbrial operon linked to long-term intestinal carriage in *Salmonella* Typhimurium (36).

Regarding siderophore systems, all the isolates had the *ent* and most of the *ybt* genes for enterobactin and yersiniabactin production, while none of them would produce aerobactin and salmochelin, frequently encoded in the large *Klebsiella* virulence plasmids (2). Moreover, all lacked the hypermucoviscosity-related gene *rmpACD*, also found in these plasmids. Regarding cell-to-cell killing capacity and toxin production, all the isolates had two to three type VI secretion systems and encoded two hemolysin-like proteins, while none had the colibactin production genes strongly associated with hypervirulent strains.

Other factors sporadically present were the Pla protease from *Yersinia pestis*, which prevents host cell apoptosis and inflammation by degrading Fas ligand (FasL) (37), and the Cah autotransporter associated with enterohemorrhagic *E. coli* infections (38). Thus, we identified an ample array of virulence factors in the Chilean *K. pneumoniae* isolates that would contribute during infection, which, however, lack the hallmark factors associated with the hypervirulent pathotype.

### Plasmids encoding carbapenemases and other resistance determinants

Forty-four plasmids among the 10 isolates were identified, then typed and clusterized using the MOB-suite tools (Table S6). Twenty-four plasmids were predicted as conjugative, 12 mobilizable, and 8 non-mobilizable. In addition, over 10 compatibility groups were observed, predominating IncR, IncFIB, IncFII, and combinations of them. Nine plasmid clusters and 18 singletons were observed, with an overall limited number of shared plasmids even among closely related isolates, except for VA04 and VA126 (CG10224), harboring three shared clusters. Moreover, intra-cluster sequence comparisons revealed substantial smaller-scale variability (Fig. S2).

**Fig 3:**
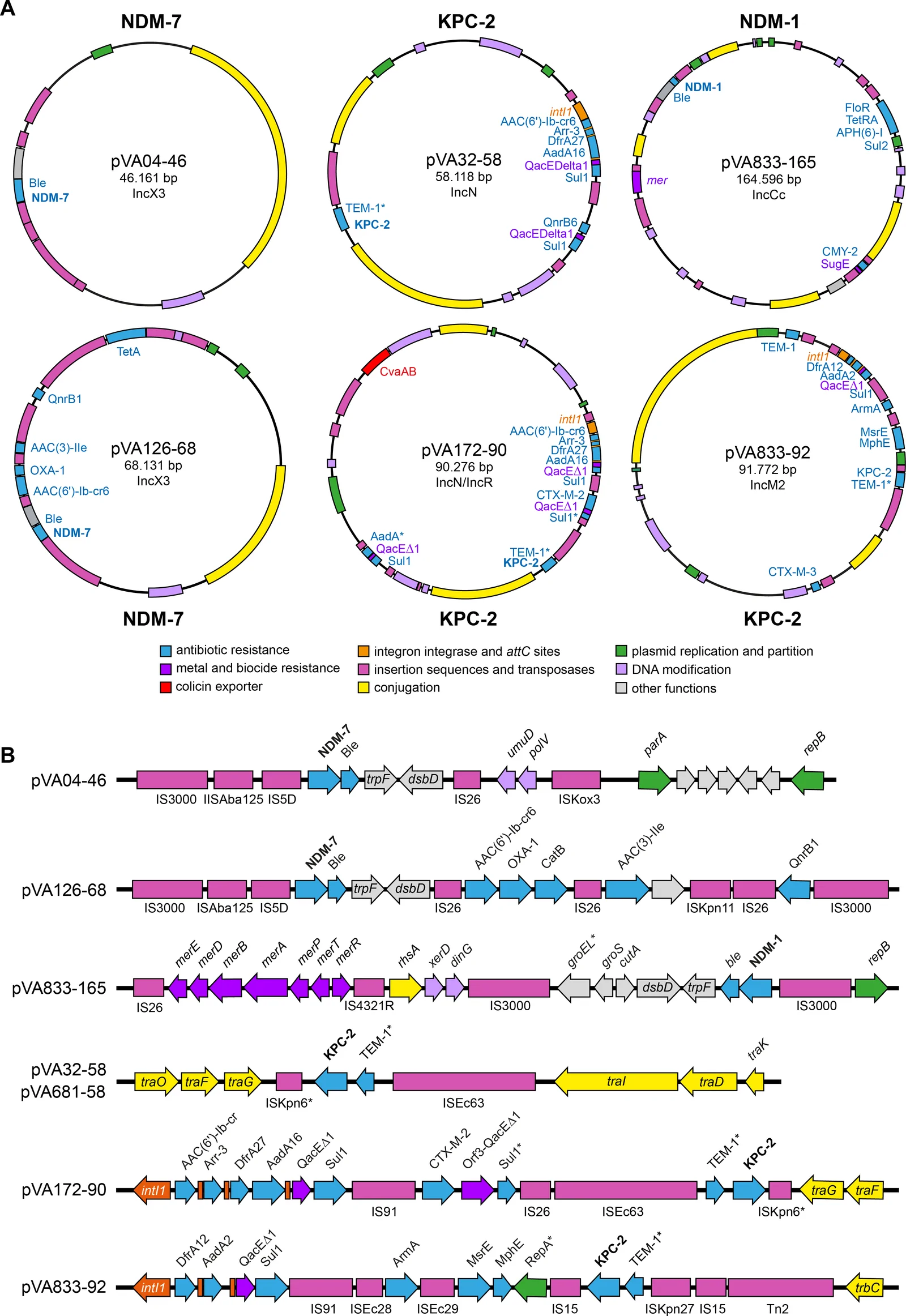
Plasmids carrying carbapenemase genes and other resistance determinants. (**A**) Schematic representation of the main NDM-1, NDM-7, and KPC-2 plasmids and their encoded functions. (**B**) Genetic context of the genes encoding carbapenemases and other resistance determinants. The annotated elements were colored as in (**A**).

Ten conjugative plasmids encoding carbapenemases were identified, four NDM-7, five KPC-2, and one NDM-1 (Fig. 3). The NDM-1 plasmid pVA833-165 (~165 kbp, IncCc, MOB cluster AA860-AJ275) showed 99% identity and 89% coverage with an uncharacterized plasmid from *Salmonella enterica* (CP009409) and ~88% coverage with an IncA/C plasmid from *K. pneumoniae* ST383 carrying *bla*_VIM_ (KR559888.1). However, pVA833-165 differed in carrying *bla*_NDM-1_ inside an IS3000-formed composite transposon, along with *bla*_CMY-2_, *floR*, *tet(A*), APH(6)-Id, *sul2*, and the mercury resistance genes *merRTPABDE* (Fig. S3A), pointing out the high-risk potential of this plasmid.

The NDM-7 plasmids pVA04-46, pVA591-46, pVA684-49, and pVA126-68 (IncX3, cluster AA038-AH615) showed ~99% identity and coverage with 46-kbp plasmids from the NCBI database, including pJN05NDM7 (NZ_MH523639.1) from uropathogenic *E. coli*, where *bla*_NDM-7_ is located in the genetic context described originally (39) (Fig. S3B). Remarkably, pVA126-68 differed from other NDM-7 plasmids in bearing an additional 21-kbp region comprising a CALIN element (clusters of attC sites lacking integron-integrases) associated with AAC(6′)-Ib-cr, *bla*_OXA-1_, *catB*, AAC(3)-IIe, and other resistance determinants (Fig. 3).

Additionally, the KPC-2 plasmids pVA172-90, pVA32-58, pVA681-58, and pVA684-37 (IncN, cluster AA552-AI756) were ~100% identical, except for the former showing additional segments. The *bla*_KPC-2_ gene was found in the NTE_KPC_-IIe genetic environment (Fig. S4A), first identified in IncN plasmids from a recent Chilean outbreak of carbapenem-resistant bacteria (e.g., pKpn-3; MT949189.1) (40) and in p33Kva16-KPC and pEC881_KPC from Colombian *E. coli* and *K. variicola* isolates (41), showing high identity with pVA681-58 and related plasmids (Fig. S4B). These KPC plasmids included a class-I integron carrying AAC(6')-Ib-cr6, *arr-3*, *dfrA27*, and *aadA16*, and a region encoding QnrB6, QacEDelta1, and Sul1. In pVA172-90, this last region was replaced by one including *bla*_CTX-M-2_ and other resistance genes. Also, it has a ~ 25 kbp extra region including *sul1*, *qacEdelta1*, and the *cvaAB* colicin exporter, among other features. Furthermore, pVA172-90 showed ≤57% coverage matches in the NCBI database. Thus, it would be a novel KPC-2 plasmid with additional resistance determinants.

A considerably different plasmid bearing *bla*_KPC-2_ was pVA833-92 (IncM2, cluster AA002-AH532), showing 97% coverage and 99% identity with pCTX-M-3 from *Citrobacter freundii* (Fig. S5A) carrying *bla*_CTX-M-3_, *bla*_TEM-1_, *aacC2*, *armA*, and an integron carrying *aadA2*, *dfrA12*, and *sul1*. Strikingly, pCTX-M-3 lacked *bla*_KPC-2_ found in pVA833-92. The inspection of the *bla*_KPC-2_ genetic environment revealed that it is immediately downstream of *bla*_TEM-1_ (truncated) and ISKpn27 (formerly ISKpn8) (Fig. S5B). Two IS26 elements that flank these three genes could have mediated the insertion of this segment into a pCTX-M-3-like plasmid. This context differed from all the known NTE_KPC_ environments (Fig. S6C) and would correspond to a novel genetic platform for mobilizing *bla*_KPC-2_, tentatively, NTE_KPC_-IIh.

We found other relevant plasmids, including pVA04-196 (AA276-AI083) carrying three metal resistance operons (*sil*, *pco*, and *ars*), *bla*_TEM-1_, *bla*_OXA-1_, and a *bla*_CTX-M-15_ (Fig. S6). pVA32-200 (AA274-AI067) also had the *sil*, *pco*, and *ars* operons, along with a wide array of antibiotic resistance genes and determinants for Klebicin B production. Additionally, we found plasmids potentially contributing to bacterial fitness during the infective process. For example, pVA684-388 (IncR, ~388 kbp) encoded determinants for K^+^ uptake, trehalose synthesis, putrescine utilization, molecular chaperones, heat shock protein metabolism, and glycerol utilization.

Next, we used MOB-recon tool to reconstruct possible plasmids present in the South American *K. pneumoniae* genomes, searching for shared clusters with plasmids described in this study. The most prevalent cluster present in 127 genomes was AA275-AI074, which along with AA274-AI067, AA276-AI083, and some other related clusters, corresponded to conjugative 170–200-kbp IncFIB plasmids carrying metal resistance (*ars*, *pco*, and *sil* operons) and variable antibiotic resistance genes. These mutually exclusive plasmids were found in 272 isolates, including seven described here, and distributed across most lineages (Fig. 1B), suggesting high transmissibility. Regarding carbapenemase plasmids, the related clusters AA552-AI746 and AA552-AI756 (IncN) were the most prevalent among those carrying *bla*_KPC-2_ (present in 90 genomes). The former was more associated with SL258, while the latter was found in diverse sublineages, including two CG340, one CG505, and one CG10224 isolates described in this study, and 23 isolated in other countries.

Conversely, the KPC-2 plasmid with the novel genetic environment, cluster AA002-AH532, was found only in VA833, while AA860-AJ275 (cluster of the NDM-1 plasmid pVA833-165) was found only in one isolate from Colombia, although we found no carbapenemase genes in this genome. Finally, the cluster AA038-AH615, corresponding to the NDM-7 plasmids described here, was present only in three more isolates from Ecuador, although no genes encoding NDM carbapenemases were found in these genomes.

### GIs encoding virulence and fitness factors

**Fig 4:**
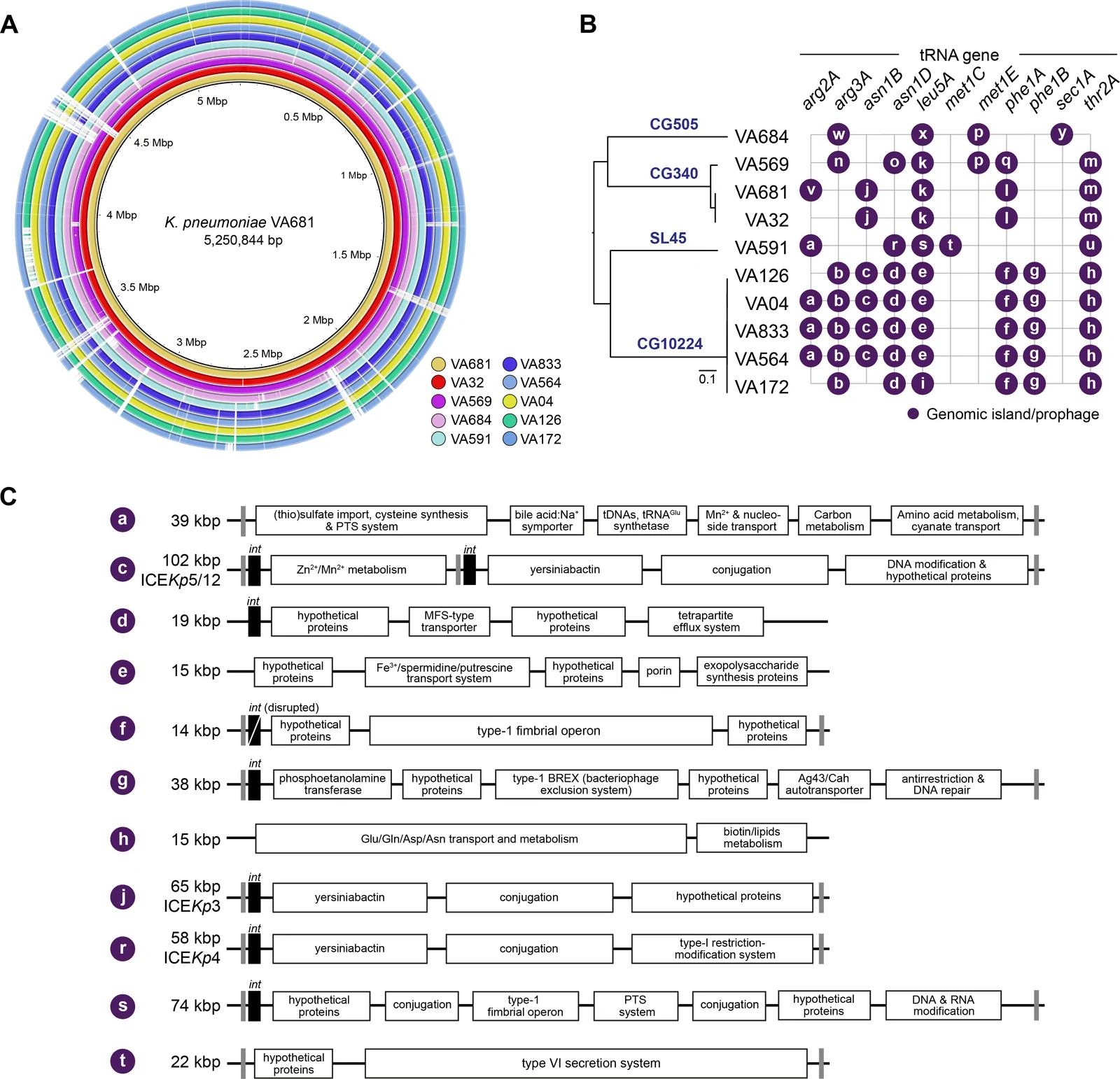
Chromosomal mobile genetic elements and encoded functions. (**A**) Chromosome sequence alignment of the Chilean isolates, as depicted by the BRIG tool. Unshared regions appear as non-colored gaps. (**B**) Mobile genetic elements integrated into tRNA genes. The letters indicate different non-redundant MGEs. The tree shown was inferred based on a cg-MSA analysis including the 629 loci of the scgMLSTv2 scheme. (**C**) Genomic islands encoding virulence and fitness factors. The gray bars indicate direct repeats; *int*, integrase.

Besides plasmids, GIs and prophages disseminate clinically relevant traits among pathogens. In this regard, the chromosome alignment of the Chilean isolates showed different variable regions (Fig. 4A), including putative MGEs integrated into transfer RNA genes (tDNAs), which act as hotspots for GI and prophage integration in *Klebsiella* and other bacteria (42).

Following a pipeline for GI and prophage prediction in the isolates, we identified 61 MGEs (24 non-redundant; id ≥95%, cov ≥90%) integrated into 11 tDNAs, with sizes ranging from ~7.5 to ~103 kbp. Furthermore, a variable chromosomal MGE content was observed even among closely related strains (i.e., CG10224 or CG340), especially in *leu5A*, *asn1B,* and *arg2A* loci, suggesting that active traffic of GIs and prophages would contribute to chromosomal variability in Chilean CR-*Kp* isolates (Fig. 4B).

After annotation, eight of the 24 non-redundant MGEs corresponded to intact, incomplete, or questionable prophages, while the rest were classified as GIs, most encoding a P4-like integrase and flanking direct repeats (Table S7). Twelve GIs encoded factors potentially linked to pathogenesis or fitness, including three members of the ICE*Kp* family (ICEKp3/5/12) integrated into asparagine tDNAs (Fig. 4C). These elements are widely disseminated *in K. pneumoniae* and carry genes for yersiniabactin production plus variable cargo modules (43). Additionally, we identified novel GIs encoding relevant functions, including carbon, amino acids, and bile acids metabolism and transport, the Cah autotransporter, fimbrial operons, a Fe3+/spermidine/putrescine transport system, and a type VI secretion system. Thus, different GIs would mediate virulence and fitness factors dissemination among Chilean CR-*Kp*.

## DISCUSSION

Genome analysis is a powerful approach to supporting pathogen surveillance, although it is still restricted in lower- and middle-income countries due to economic limitations and less expeditious access to equipment and supplies (44). Regrettably, this causes many geographical regions, including most of South America, to be underrepresented in genome databases and, thus, elusive in the global epidemiological landscape. In this line, we used genomic analyses to unveil the resistance and virulence mechanisms of Chilean CR-*Kp*, focusing on plasmids and other mobile elements disseminating these factors. Moreover, we revisited the genomic landscape of South American *K. pneumoniae*, identifying distinctive and shared features with the Chilean isolates.

Although a limited number of strains were sequenced due to the abovementioned limitations, they provided relevant baseline knowledge for further local and global studies on *K. pneumoniae* epidemiology. Moreover, although the sequenced isolates were from only three out of 16 regions, Chile is a highly centralized country, and roughly 90% of the total isolates collected as part of the national surveillance came from these regions. Therefore, additional studies with more isolates covering less represented regions would provide a more representative view of the country. Likewise, more sequenced genomes from South American countries currently absent from the databases would provide a more representative continental panorama.

Although most CR-*Kp* infections worldwide were attributed mainly to SL258 and a few other lineages, the burden of local problem clones remains substantial (2). CG258 and CG340 KPC-producing *Kp* have been widely reported in South America, although many less frequent lineages, normally defined at ST level, were also found (4, 45 – 47). In Chile, reported CR-*Kp* mainly belong to ST11, ST258, ST29, ST1588, and ST1161 (proposed to be native to Chile) (48, 49). Conversely, ST25, linked to a relatively low number of CR-*Kp* infections worldwide, predominated in this study. Phylogenomic analyses revealed they belonged to CG10224 and were highly similar to four Chilean genomes from ST25 strains isolated in 2012, 2013, and 2017, which, however, lacked carbapenemases genes. The 2017 strain was isolated from a neutropenic patient with a recalcitrant bloodstream infection, which evolved toward full carbapenem resistance despite not producing carbapenemases (50). Thus, the more recent acquisition of *bla*_KPC-2_, *bla*_NDM-1_, and *bla*_NDM-7_ plasmids by this clone in Chile, especially the KPC-2/NDM-7 combination as in VA833, or the evolution of colistin-resistant isolates such as VA172, is of special concern. Furthermore, all the CG10224 isolates described in this study came from invasive infections, three from the bloodstream, one from cerebrospinal fluid, and one from bone tissue. These findings stress this lineage’s rapid evolution and potential risk.

ST25 *Kp* causing invasive infections were also reported in Argentina (51) and in an outbreak in Ecuador (52). As in this study, these ST25 isolates showed a K2 capsular serotype linked to increased virulence (2), though no genomic data is available for comparison. Although two draft genomes associated with infections in Argentina proposed to be ST25 were reported (53), Kleborate analysis indicated that they are ST629 and ST551-1LV. On the other hand, among our South American data set, we found one CG10224 KPC-2-producing strain isolated from a bloodstream infection in Argentina in 2015, and another in Colombia in 2014. Thus, ST25 K2 CR-*Kp* seems to be an emerging clone in South America, where carbapenem resistance and increased virulence converge. However, more genomes for comparative analyses are required to examine the presence of CG25 vs. CG10224 and if these clones show different epidemiological features.

Additionally, we identified two isolates from the less frequent ST45 and ST505. ST45 is an emerging carbapenemase-producing clone highly prevalent in the EuSCAPE surveillance study (15, 54). Also, ST45 KPC-2-producing *Kp* was reported in Venezuela (55), while infections of ST45 *Kp* lacking carbapenemases were reported in Brazil and Mexico (56, 57). Thus, to our knowledge, VA591 corresponds to the first report of ST45 *Kp* producing NDM-7 in South America, which also showed resistance to colistin and diverged from other ST45 isolated worldwide, corresponding to a CG not described previously. On the other hand, very few studies described ST505 *Kp* infections, including one from Mexico (56), although these isolates were carbapenem-sensitive. We found no previous reports of ST505 CR-*Kp,* and VA684 was the only ST505 isolate from our genome database producing carbapenemases. Thus, this would be the first report of an ST505 *Kp* producing both NDM-7 and KPC-2.

The high levels of resistance found in the isolates were correlated with an ample array of acquired resistance genes, some of which were found with relatively low frequency in other *K. pneumoniae* isolates and were not previously reported in Chile, including *bla*_NDM-7_, alone or combined with *bla*_KPC-2_, *bla*_SHV-31_, and *bla*_CMY-2_ (first report in *K. pneumoniae*). Moreover, most isolates also carried genes conferring resistance to metals and disinfectants, in agreement with previous reports showing their co-selection with antibiotic resistance genes, widening the toolbox to resist antimicrobial chemotherapy and persist in clinical, urban, and polluted environments (58, 59).

We identified numerous plasmids and GIs among a relatively small number of Chilean isolates, anticipating a remarkable diversity of these elements in the *K. pneumoniae* population. The sequencing of additional genomes, especially from underrepresented geographical areas, will contribute to unveiling this yet-to-be-known MGE diversity. Most of these MGEs included resistance and virulence genes, but also genes potentially involved in cell fitness, whose contribution to host infection deserves further studies. Moreover, plasmid reconstruction in the South American data set revealed that several of the plasmids found in the isolates described in this study would be absent in other strains.

Plasmids carrying carbapenemase genes, especially *bla*_KPC_, are the main drivers of carbapenem resistance in *Enterobacteriaceae* globally (60). Although *bla*_KPC_ genes were initially associated with a ~10 kb Tn4401 derivative, novel non-Tn4401 elements (NTE_KPC_) for their dissemination have been described (60). Both platforms were described in a previous study in Chile (48), with the predominance of the so-called Argentinian variant 1a (61) (NTE_KPC_-IIa) in non-conjugative plasmids from *K. pneumoniae* ST11, ST29, and ST1161. Conversely, in this study, pVA32-58 and related conjugative plasmids (IncN) from ST25, ST11, and ST505 isolates had the *bla*_KPC-2_ gene in the NTE_KPC_-IIe genetic environment, which recently emerged in Chile in an ST11 *K. pneumoniae* (and other *Enterobacteriaceae*) and in *K. variicola* in Colombia (40, 41). Moreover, we found these plasmids and related variants highly prevalent among the South American genomes and thus would be one of the leading mobile elements mediating *bla*_KPC-2_ acquisition by *K. pneumoniae* in this region. Besides, to our knowledge, pVA833-92 corresponds to the first report where *bla*_KPC-2_ jumped to a pCTX-M-3-like IncM2 plasmid, located in a novel class II KPC genetic environment flanked by IS26 elements named NTE_KPC_-IIh.

Half of the isolates studied here encoded an NDM carbapenemase, with two also encoding *bla*_KPC-2_. In Chile, *K. pneumoniae* producing NDM-1 was first reported in 2018 in an ST1588 isolate (49). The novel *bla*_NDM-1_ plasmid pVA833-165 (IncC) from an ST25 isolate had a known IS3000 environment but differed from known plasmids in carrying *bla*_CMY-2_ and several other antibiotic and metal resistance genes. Meanwhile, the *bla*_NDM-7_ plasmids (IncX3) had a Tn125 environment, where pVA126-68 differed from previously known plasmids in having an additional 21-kbp region encoding several additional resistance genes.

The genome-based virulence profiling revealed several known factors linked to cell adhesion, iron uptake, and cell-to-cell killing. Also, other factors not described before in *K. pneumoniae,* such as the Cah autotransporter from enterohemorrhagic *E. coli* involved in autoaggregation and biofilm formation, and the *Y. pestis* Pla protease involved in signaling to manipulate host cell death and inflammation. Further studies are required to investigate the role of these factors in *K. pneumoniae* pathogenesis. In this line, none of the isolates described in this study showed the typical markers of hypervirulent strains associated with pyogenic liver abscesses and other severe community-acquired infections, mainly in Southeast Asia (typically from CG23). However, a recent report described the isolation in Chile of KPC-2-producing CG23 *K. pneumoniae* carrying the virulence plasmid and other features of hypervirulent strains (62). This finding and the potential emergence of other high-risk clones, such as carbapenemase-producing CG10224, stress the importance of introducing genomic tools to reinforce CR-*Kp* surveillance, providing valuable information to help anticipate and mitigate outbreaks.

## Data Availability

The complete genome sequences of the isolates described in this study were deposited in the NCBI Genome database under the BioProject accession PRJNA815754. Additionally, the individual accession numbers for each isolate (chromosome and plasmids) are indicated in Table 2. The accession numbers of the *K. pneumoniae* assemblies included in the global and South American data sets described here can be found in Tables S3 and S4.

## SUPPLEMENTAL MATERIAL

Supplementary Figures and Tables spectrum.00399-23-s0001.pdf Supplementary Figures and Tables not provided as spreadsheets.

Table S3 spectrum.00399-23-s0002.xlsx Supplementary Table 3. Accessions and cgLIN codes of the 10,810 *K. pneumoniae* genomes included in the global dataset used in this work.

Table S4 spectrum.00399-23-s0003.xlsx Supplementary Table 4. Kleborate results and cgLIN codes for the South American *K. pneumoniae* genome set.

Table S5 spectrum.00399-23-s0004.xlsx Supplementary Table 5. Harmonized list of antibiotic resistance genes identified in the Chilean *K. pneumoniae* isolates described in this study.

Table S6 spectrum.00399-23-s0005.xlsx Supplementary Table 6. Main features of the plasmids identified in the *K. pneumoniae* isolates described in this study.

Table S7 spectrum.00399-23-s0006.xlsx Supplementary Table 7. tDNA-associated mobile genetic elements found in the chromosome of the *K. pneumoniae* isolates described in this work.

ASM does not own the copyrights to Supplemental Material that may be linked to, or accessed through, an article. The authors have granted ASM a non-exclusive, world-wide license to publish the Supplemental Material files. Please contact the corresponding author directly for reuse.
