# Supplementary material for: Antimicrobial resistance, pathogenic potential, and genomic features of carbapenem-resistant Klebsiella pneumoniae isolated in Chile: high-risk ST25 clones and novel mobile elements
Source: Microbiol Spectr. 2023 Sep 14;11(5):e00399-23. doi: 10.1128/spectrum.00399-23 (PMC10581085; doi:10.1128/spectrum.00399-23)
Supplement: Supplementary Figures and Tables — not provided as spreadsheets. [file spectrum.00399-23-s0001.pdf]

SUPPLEMENTARY MATERIAL

Antimicrobial resistance, pathogenic potential, and genomic features of carbapenem-resistant *Klebsiella pneumoniae* isolated in Chile: high-risk ST25 clones and novel mobile elements

Marcelo Veloso[1], Patricio Arros[1], Joaquín Acosta[1], Camilo Berríos-Pastén[1], Roberto Rojas[1], Macarena Varas[1], Miguel L. Allende[3], Francisco P. Chávez[2], Pamela Araya[4], Juan Carlos Hormazábal[4], Rosalba Lagos[1], Andrés E. Marcoleta[1,*]

1Grupo de Microbiología Integrativa, Laboratorio de Biología Estructural y Molecular BEM, Departamento de Biología, Facultad de Ciencias, Universidad de Chile. Santiago, Chile.
2Laboratorio de Microbiología de Sistemas, Departamento de Biología, Facultad de Ciencias, Universidad de Chile. Santiago, Chile.
3Millenium Institute Center for Genome Regulation, Facultad de Ciencias, Universidad de Chile. Santiago, Chile.
4Instituto de Salud Pública. Santiago, Chile.

*corresponding author

Supplementary Table 1. *K. pneumoniae* isolates studied in this work.

Isolate	Sex	Age	Collection date	Source	Region of origin
VA4	M	36	05-12-2018	Cerebrospinal fluid	Metropolitan
VA32	F	81	04-01-2019	Bronchoalveolar lavage	Metropolitan
VA126	M	33	16-01-2019	Blood	Metropolitan
VA172	M	71	25-01-2019	Bone tissue	Maule
VA564	M	79	02-04-2019	Blood	Los Lagos
VA569	M	53	06-04-2019	Peritoneal fluid	Metropolitan
VA591	M	29	11-04-2019	Blood	Metropolitan
VA681	F	32	24-04-2019	Abscess	Metropolitan
VA684	F	15	25-04-2019	Catheter blood	Metropolitan
VA833	F	54	23-05-2019	Blood	Metropolitan

Supplementary Table 2. Phenotypic tests for carbapenemase production and beta-lactamase gene detection in the *K. pneumoniae* isolates studied in this work.

Isolate	Blue Carba	Boronic Acid	carbapenemase gene	ESBL
VA4	+	-	NDM+	+
VA32	+	+	KPC+	+
VA126	+	-	NDM+	+
VA172	+	+	KPC+	+
VA564	-	-	-	+
VA569	-	-	-	+
VA591	+	-	NDM+	+
VA681	+	+	KPC+	+
VA684	+	+	KPC+/NDM+	+
VA833	+	+	KPC+/NDM+	+

Supplementary Table 3. Accessions and cgLIN codes of the 10,810 *K. pneumoniae* genomes included in the global dataset used in this work. (Provided as a separate spreadsheet).

Supplementary Table 4. Kleborate results and cgLIN codes for the South American *K. pneumoniae* genome set. (Provided as a separate spreadsheet).

Supplementary Table 5. Harmonized list of antibiotic resistance genes identified in the Chilean *K. pneumoniae* isolates described in this study. (Provided as a separate spreadsheet).

Supplementary Table 6. Main features of the plasmids identified in the *K. pneumoniae* isolates described in this study (Provided as a separate spreadsheet).

Supplementary Table 7. tDNA-associated mobile genetic elements found in the chromosome of the *K. pneumoniae* isolates described in this work (Provided as a separate spreadsheet).

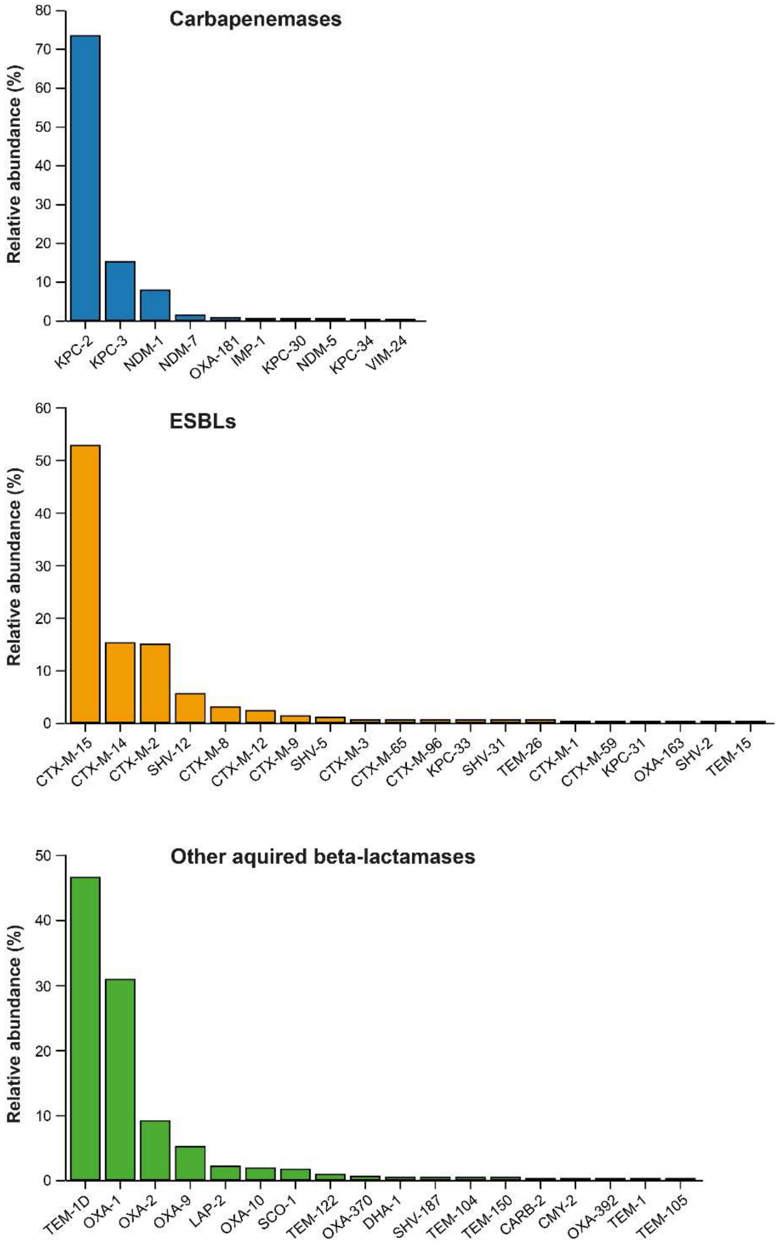

Supplementary Figure 1. Prevalence of genes encoding carbapenemases, ESBLs, and other acquired beta-lactamases found in the 602 South American *K. pneumoniae* genome dataset, according to Kleborate prediction. The prevalence was determined as the total number of genomes showing each beta-lactamase divided by the total number of genomes. Subvariants detected by kleborate (e.g., OXA-1* or TEM-1D.v1^), were counted as part of the more general category (e.g., OXA-1 and TEM-1).

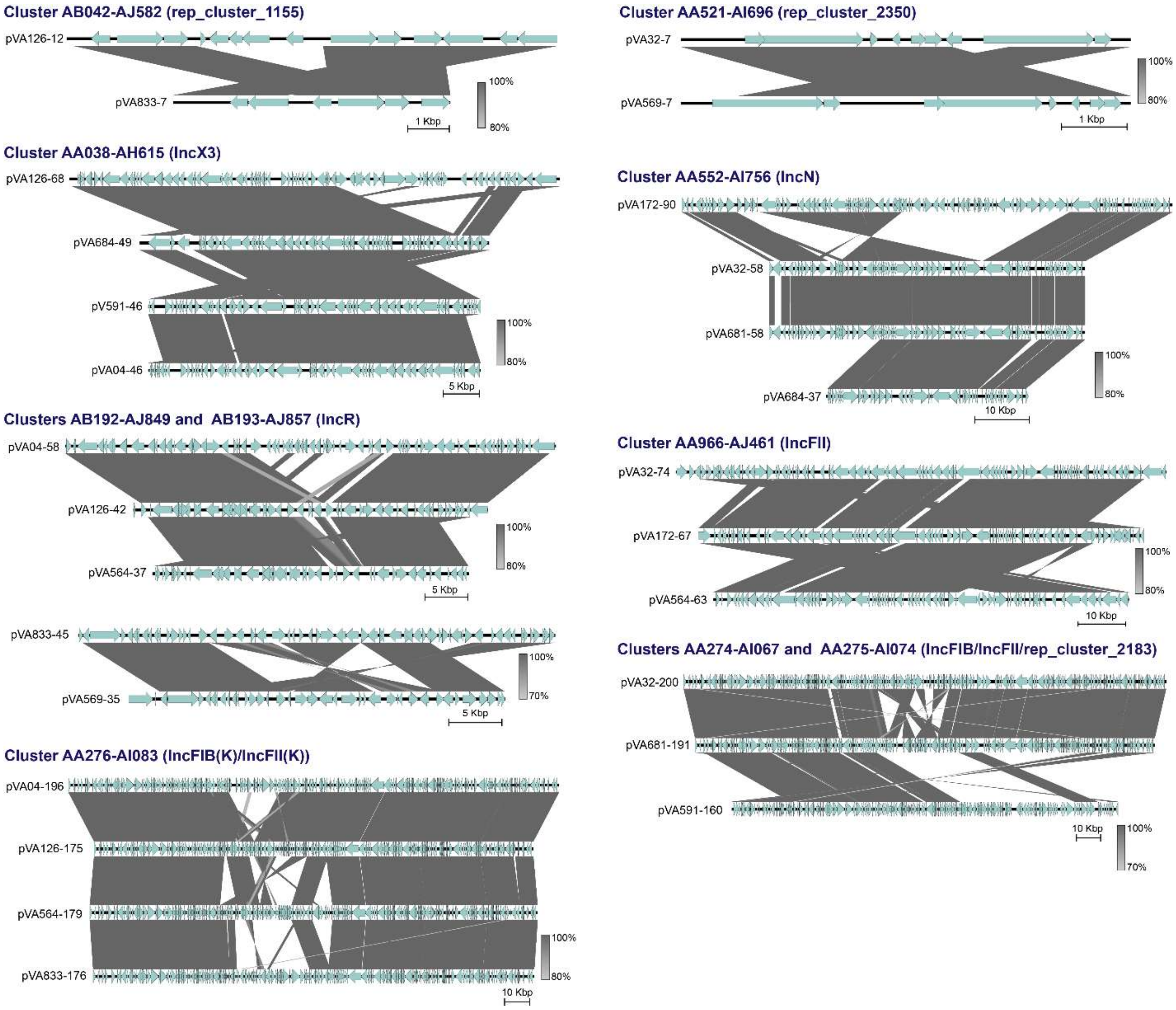

Supplementary Figure 2. Graphical representation of the sequence alignment among plasmids from the Chilean CR-*Kp* isolates described in this study, grouped according to the different clusters identified through MOB-suite analysis. The alignment plots were generated using the EasyFig tool.

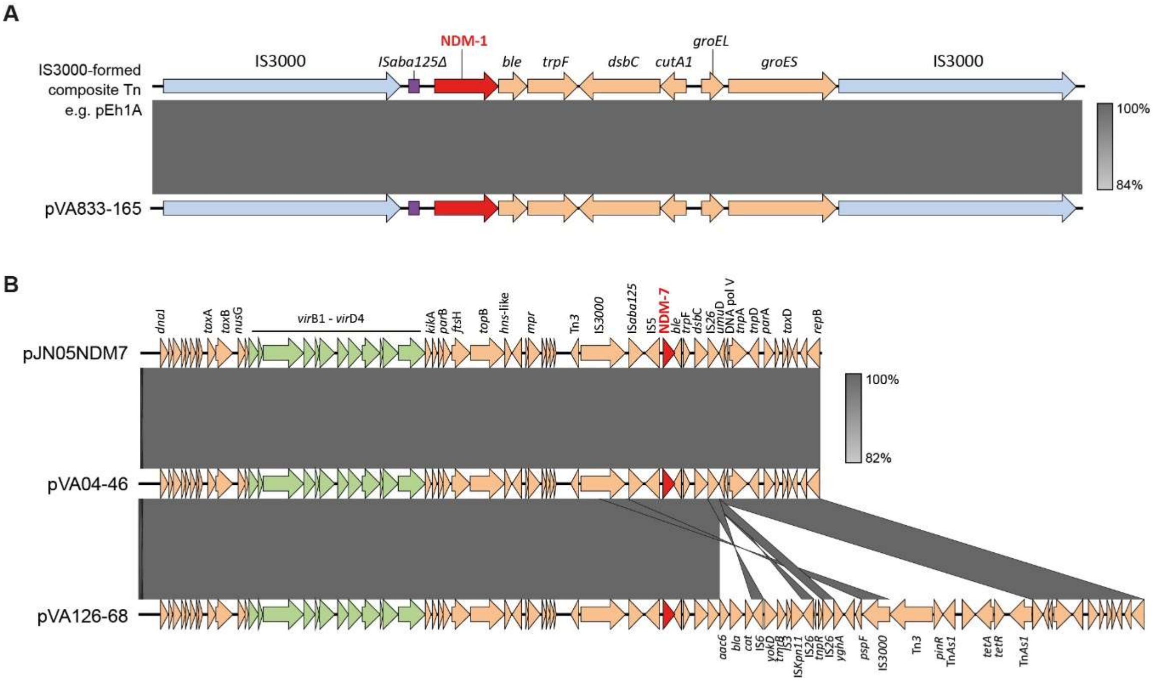

Supplementary Figure 3. Genetic context of the *bla*$_{\text{NDM-1}}$ (A) and *bla*$_{\text{NDM-7}}$ (B) genes encoded in plasmids from the Chilean CR-*Kp* isolates described in this study. pEh1A accession: KR822246; pJN05NDM7 accession: MH523639. The alignment plots were generated using the tool EasyFig.

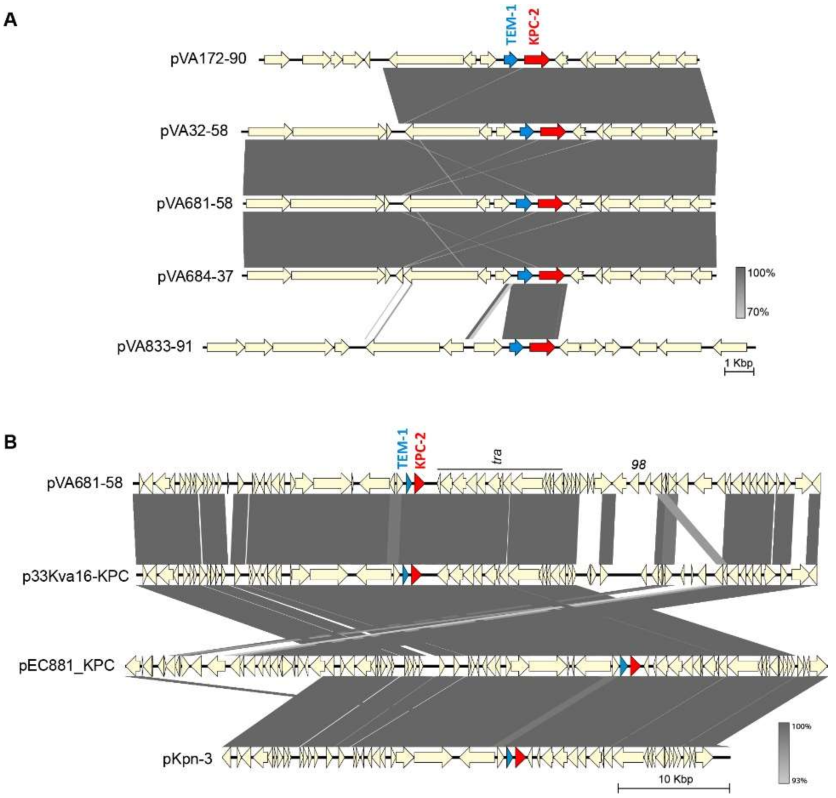

Supplementary Figure 4. (A) Comparison of the $\mathit{bla}_{\text{KPC-2}}$ genetic context found in different plasmids from the Chilean CR-Kp isolates described in this study. (B) Comparison of the $\mathit{bla}_{\text{KPC-2}}$ genetic context found in pVA681-58 and related plasmids described previously bearing the NTE_{KPC}-IIe context.

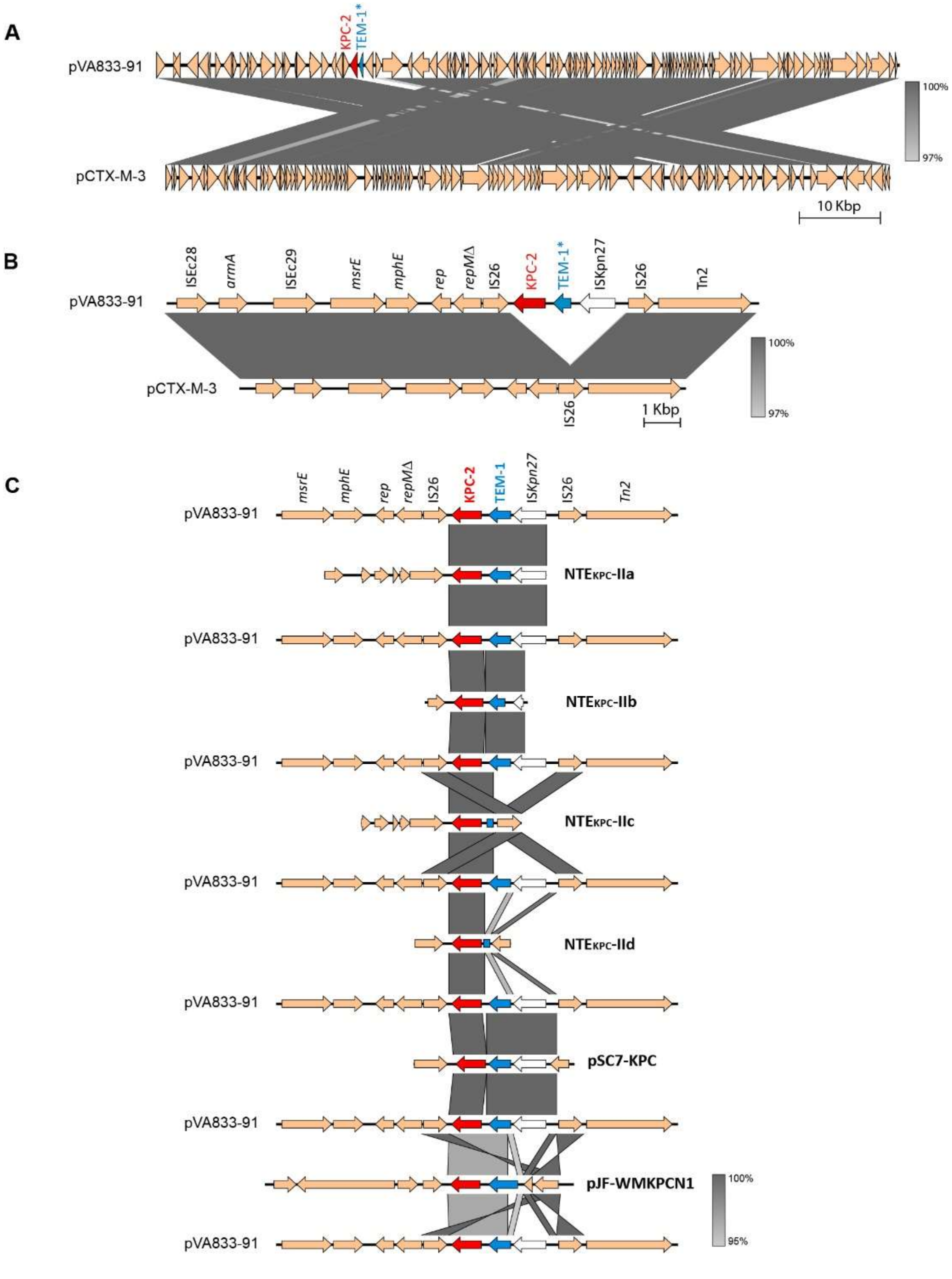

Supplementary Figure 5. (A) Sequence alignment between pVA833-91 and pCTX-M-3 sharing high identity. (B) Comparison of the *bla*$_{KPC-2}$ genetic context in pVA833-91 and the corresponding region in pCTX-M-3 lacking this carbapenemase gene. (C) Comparison of the *bla*$_{KPC-2}$ genetic context found in pVA833-91 and other class-II contexts (known as NTE$_{KPC}$-II) reported previously for this gene [38], indicating that this would correspond to a novel genetic environment for *bla*$_{KPC-2}$. pSC7-KPC (accession NZ_CP030267) and pJF-WMKPCN1 (accession NZ_KX881941) are plasmids recently proposed to host novel unclassified contexts.

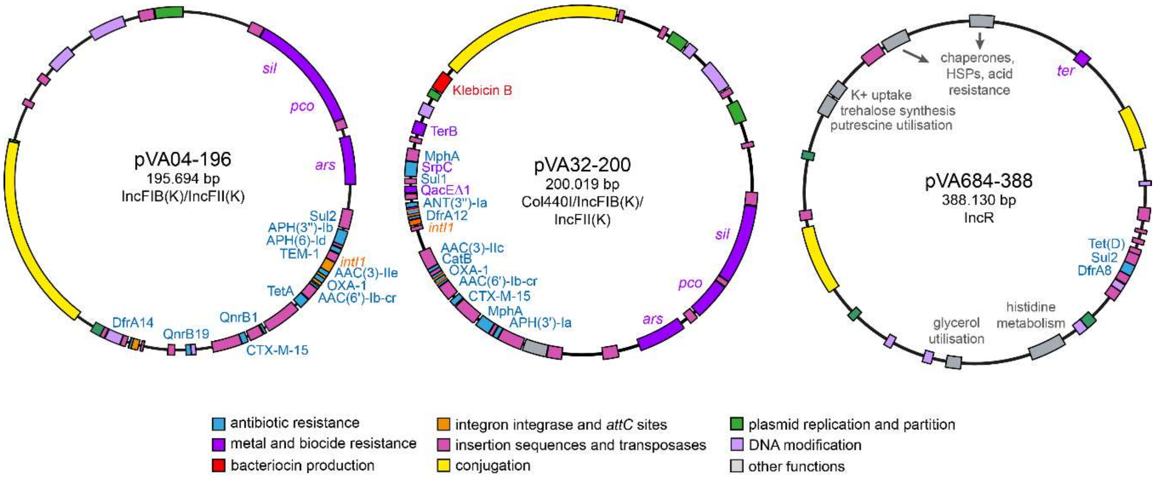

Supplementary Figure 6. Plasmids encoding different factors which could contribute to bacterial resistance, virulence and fitness during host infection, found in the CR-Kp isolates described in this study.
